# Supplementary material for: Innate connectivity patterns drive the development of the visual word form area
Source: Sci Rep. 2020 Oct 22;10:18039. doi: 10.1038/s41598-020-75015-7 (PMC7582172; doi:10.1038/s41598-020-75015-7)
Supplement: Supplementary file 1 — Supplementary Information [file 41598_2020_75015_MOESM1_ESM.pdf]

Title: Innate connectivity patterns drive the development of visual word form area

Jin Li[1]*, David E. Osher[1], Heather A. Hansen[1], Zeynep M. Saygin[1]*

[1]Department of Psychology, The Ohio State University, Columbus, OH
*Correspondence to: li.9361@osu.edu and saygin.3@osu.edu

Supplementary Information

Supplementary inventory:

Supplementary Results 1&2: Using four fMRI tasks, subject-specific functional regions (fROIs) were identified in an independent group of adult subjects within the functional parcels used in the main analyses. fROIs across subjects demonstrate variability in spatial location across subjects and demonstrate the need for larger parcel regions that will certainly encompass the sites of functional specificity in the neonates in the main study. Percent signal change was also extracted in independent runs to demonstrate functional specificity of these fROIs.

Supplementary Results 3: Two-way mixed design ANOVA of language regions' FC using size as a covariate and two-way mixed design ANOVA of VWFA's FC using size as a covariate.

Supplementary Results 4: FC between VWFA and temporal regions, and VWFA and frontal regions.

Supplementary Results 5: FC results for new Neurosynth-overlapped parcels.

Supplementary Results 6: Comparison between registration accuracy of ANTs and FLIRT.

Supplementary Results 7: Replication of the main results using the VWFA-p that is restricted to the posterior proportion of the VWFA parcel.

Supplementary Figure 1: Probabilistic maps created based on fROIs across a group of adults (independent of those in the main study).

Supplementary Figure 2: Functional response profiles from separate runs than those used to define the fROIs in these independent adult subjects.

Supplementary Figure 3: Average voxel-wise functional connectivity maps within the ventral temporal cortex (VTC) using language regions as the seed.

Supplementary Figure 4: FC between VWFA (seed) and temporal and frontal regions.

Supplementary Figure 5: Voxel-wise analyses from VWFA to frontotemporal cortices.

Supplementary Figure 6: Averaged whole-brain functional connectivity maps of VWFA for adults and neonates.

Supplementary Figure 7: FC between language (seed) and visual regions and FC between VWFA (seed) and temporal and frontal regions with new Neurosynth-overlapped parcels.

Supplementary Figure 8: Registration results.

Supplementary Figure 9: FC results using VWFA-p

Supplementary Table 1: Information of parcels used in the current study.

Supplementary Table 2&3: t test results for the VWFA-p.

Supplementary Results 1. We applied functional parcels to an independent group of adults (different individuals than adults involved in the analyses below; see **Methods**). Participants were run on the VWFA, Language & Speech, MD, and Dynamic localizers, which are the same or similar fMRI localizers as those used in the previous studies from which we drew the functional parcels in the main analyses; see **Methods** and **Supplementary Table 1**. Experimental parameters were similar or identical to those used in the published studies for each localizer. Briefly, participants viewed the VWFA localizer where they saw words, line-drawings of faces, and objects (details in Saygin et al. (2016)[1] and 3 additional fMRI localizers: the Language & Speech localizer with auditory stimuli consisting of English sentences, sentences with similar prosody but constructed from nonsense words, and statistically-matched textured speech which controlled for low-level auditory features but did not have recognizable speech sounds[2-4]; the MD localizer with Hard and Easy task conditions of a spatial working memory paradigm[5]; and the Dynamic localizer consisting of movie clips of faces, bodies, objects, and scenes[6, 7]. Data were collected on a 3T Siemens scanner with a 32-channel head-coil. Acquisition parameters were similar or identical to those used in the published studies for each localizer: the VWFA localizer was acquired with $2mm^3$ resolution, 2s TR, 30ms TE, 90° flip, 100x100 base resolution, 25 slices approximately parallel to the base of the temporal lobe to cover the entire inferior temporal cortex. We additionally collected a field map for distortion correction with the same slice prescription as the fMRI sequence (25 slices, $2mm^3$ resolution, 500ms TR, 55° flip, 100x100 base resolution). The Language & Speech and MD localizers were acquired with 2s TR, 30ms TE, 90° flip, GRAPPA acceleration factor 2, 2x2mm in-slice resolution, 4mm slice thickness, 31 slices for full-brain coverage; and the Dynamic localizer was acquired with 2TR, 30ms TE, 90° flip, $3mm^3$ voxels, and 32 slices for full-brain coverage. Experiments were counter-balanced across participants. A high-resolution (1 mm^3) three-dimensional magnetization-prepared rapid acquisition with gradient echo (MPRAGE) scan was also acquired in all participants. All the imaging data were analyzed using standard pre-processing steps with Freesurfer (www.surfer.nmr.mgh.harvard.edu/) and FsFast (www.surfer.nmr.mgh.harvard.edu/FsFast/). Images were motion-corrected, de-trended, and fit using a standard gamma function (d = 2.25 and t = 1.25). Runs were registered to each subject’s anatomical image using Freesurfer’s bbregister.

We applied the functional parcels used in the main analyses to identify subject-specific functional regions (fROIs) using the same GSS approach and contrasts as outlined in the **Methods**. Individual fROIs were defined using the top 10% most active voxels within these parcels for each contrast of interest: words > line-drawings of objects for the VWFA, line-drawings of faces > objects for face regions, English > nonword sentences for language regions, nonword sentences > texturized speech for speech region, hard > easy for MD regions, movie clips of scenes > objects for scene regions and objects > scrambled objects for object regions. Each subject's fROI was registered to Freesurfer's CVS_MNI152 standard space and probabilistic heat maps were created based on these adults (shown in **Supplementary Figure 1**). The figure shows that these functionally selective fROIs are somewhat consistent across participants, where more than half of subjects' (>=7) fROIs land within similar spatial locations; however, we also observed large inter-subject variability as evidenced by the large spread of fROIs across e.g. temporal cortex for temporal language fROIs across subjects. These fROIs demonstrate variability in spatial location across subjects and demonstrate the need for larger parcel regions that will certainly encompass the sites of functional specificity in the neonates in the main study.

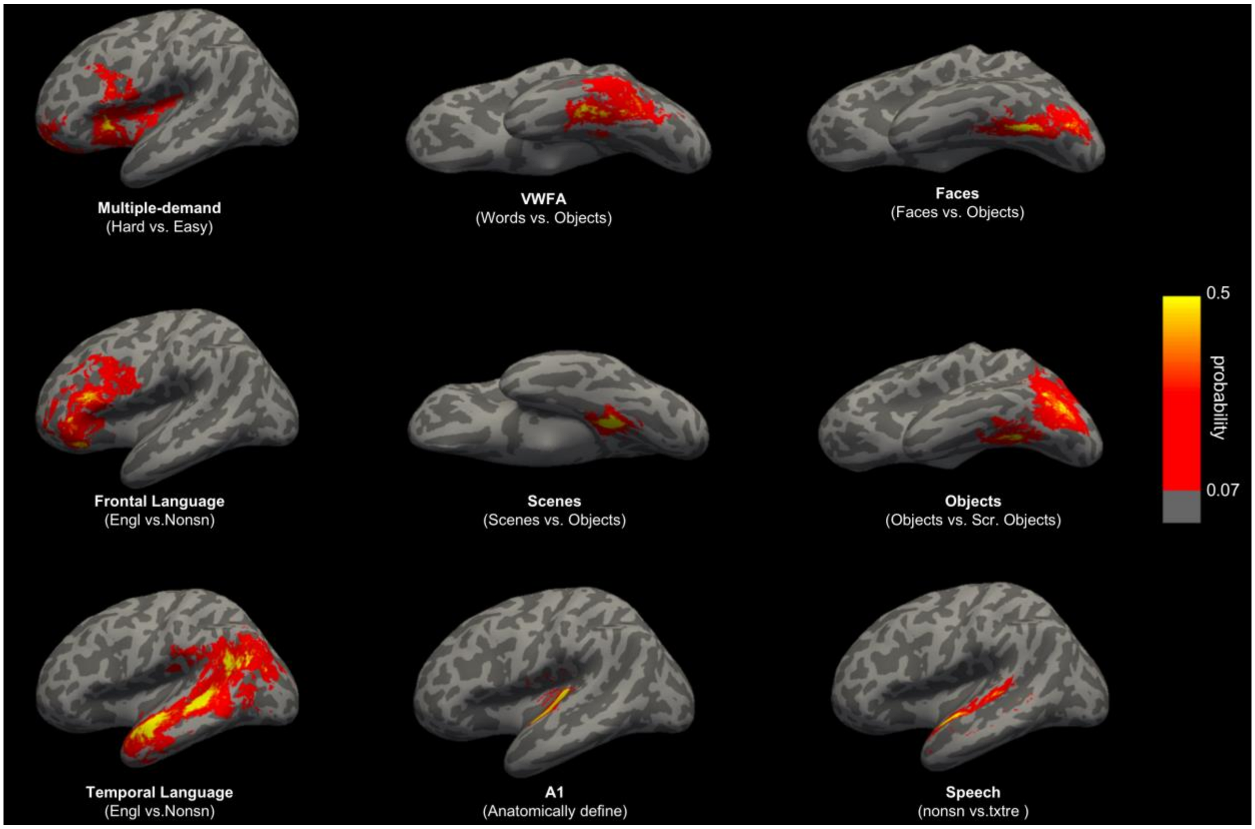

Supplementary Figure 1 | Probabilistic maps created based on subject-specific functional regions. Heatmaps show voxels that show functional selective response to a given contrast in at least 1 subject (prob. = 0.07) to equal or greater than 6 subjects (prob. = 0.5). Contrasts of interest for each region are provided in the text and below each region; note that A1 for each individual was anatomically defined in Heschl's gyrus (superior and transverse temporal cortex from the FreeSurfer Desikan-Killiany parcellation). Engl, English sentences; Nonsn, non-word sentences; txtre, texturized; Src. Objects, scrambled objects. These fROIs demonstrate variability in spatial location across subjects and demonstrate the need for larger parcel regions that will certainly encompass the sites of functional specificity in the neonates in the main study.

Supplementary Results 2. Additionally, we extracted percent signal change (PSC, from independent fMRI runs from those used to define the fROIs) for the different experimental conditions across the different localizers to demonstrate functional selectivity of responses. fROI PSCs were averaged for each category of interest and nonparametric Wilcoxon sign-rank tests were performed to see if the conditions of interest for each fROI were higher than all of the other conditions (i.e. one test was performed for each fROI). **Supplementary Figure 2** shows functional response profiles for fROIs that were identified within the functional parcels, and demonstrates distinct response patterns across fROIs. With the exception of frontal lang and MD which showed trending effects, all other fROIs showed significantly higher responses to the mental function they are posited to be selective to vs. other conditions ($p<0.05$, Bonferroni-Holm corrected). Altogether, Supplementary Figures 1 and 2 suggest that the functional parcels used in the main results will capture both functionally specific responses to the functions of interest tested in the present study and also tolerate variability between individuals.

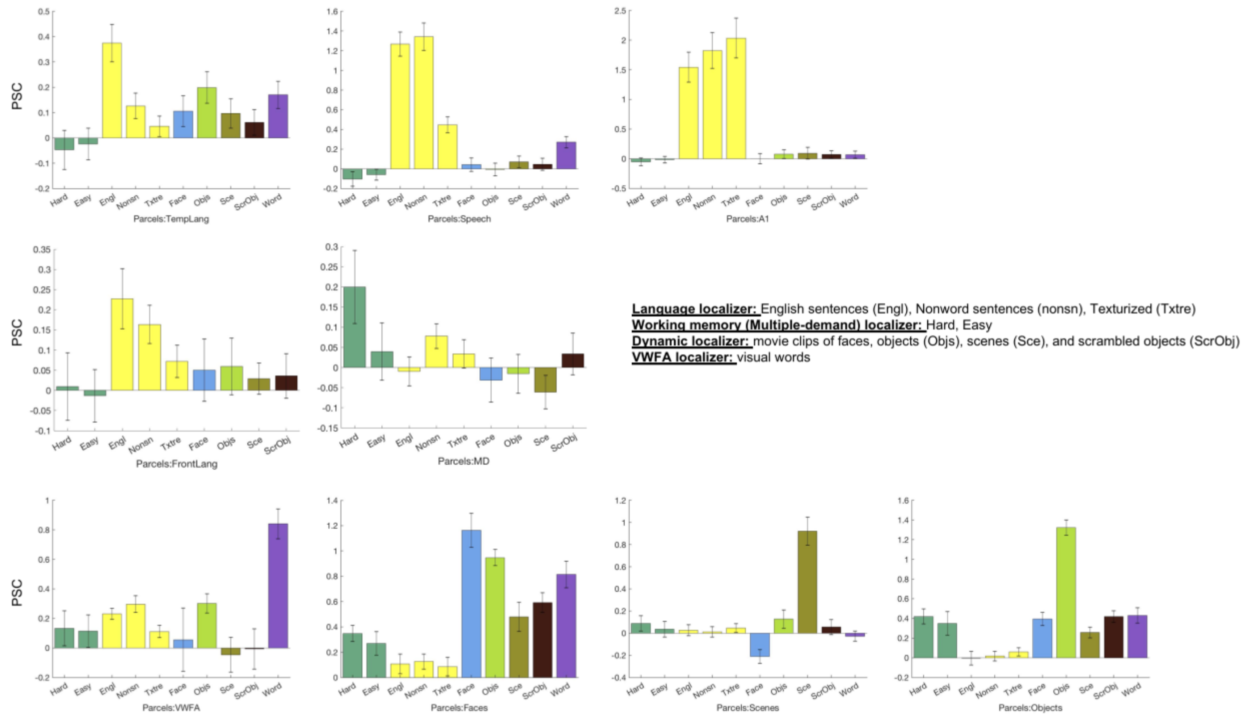

Supplementary Figure 2 | Functional response profiles for every fROI. Percent signal change (PSC) was extracted from independent run (separate from those used to define each region) for every functional localizer. Note that the field-of-view (FOV) of the VWFA localizer only had temporal cortex coverage so the response to the word condition is not shown for frontal language and MD regions. Each fROI shows selective responses to the conditions of interest vs. other conditions across fMRI experiments (temporal

lang: Engl > others: p=0.0012, W=98; Frontal lang: p=0.052, W=79; Speech: Nonsn, Engl > others: p<0.0001, W=105; A1: Engl, Nonsn,Txtre > others: p<0.0001, W=105; MD: Hard > others: p=0.067, W=77; Faces: faces > others: p<0.0001, W=105; Objects: Objects > others: p<0.0001, W=105; Scenes: scenes > others: p<0.0001, W=105; Words: words > others: p=0.0002, W=78.

Supplementary Results 3. We performed a two-way mixed design ANOVA of language regions' FC with age group (neonate, adult) as the between-group variable and target (VWFA, faces, scenes, objects) as the within-group variable, and the size of the functional parcels as a covariate. We found that even after accounting for size, the main effects of target ($F(3,311) = 7.28$, $p < 0.001$, partial $\eta^2 = 0.07$), group ($F(1,311) = 7.49$, $p = 0.007$, partial $\eta^2 = 0.02$), and the interaction ($F(3, 311) = 5.65$, $p = 0.001$, partial $\eta^2 = 0.05$) were still significant. We also performed a two-way mixed design ANOVA with age group (neonate, adult) as the between-group variable and target (language regions, multiple-demand regions, speech, A1) as the within-group variable, and the size of the functional parcels as a covariate. Our results showed no main effect of age group ($F(1,311) = 3.37$, $p = 0.067$, partial $\eta^2 = 0.01$), significant main effect of target ($F(3,311) = 3.1$, $p = 0.027$, partial $\eta^2 = 0.03$), and no significant interaction ($F(3,311) = 0.68$, $p = 0.566$, partial $\eta^2 = 0.01$).

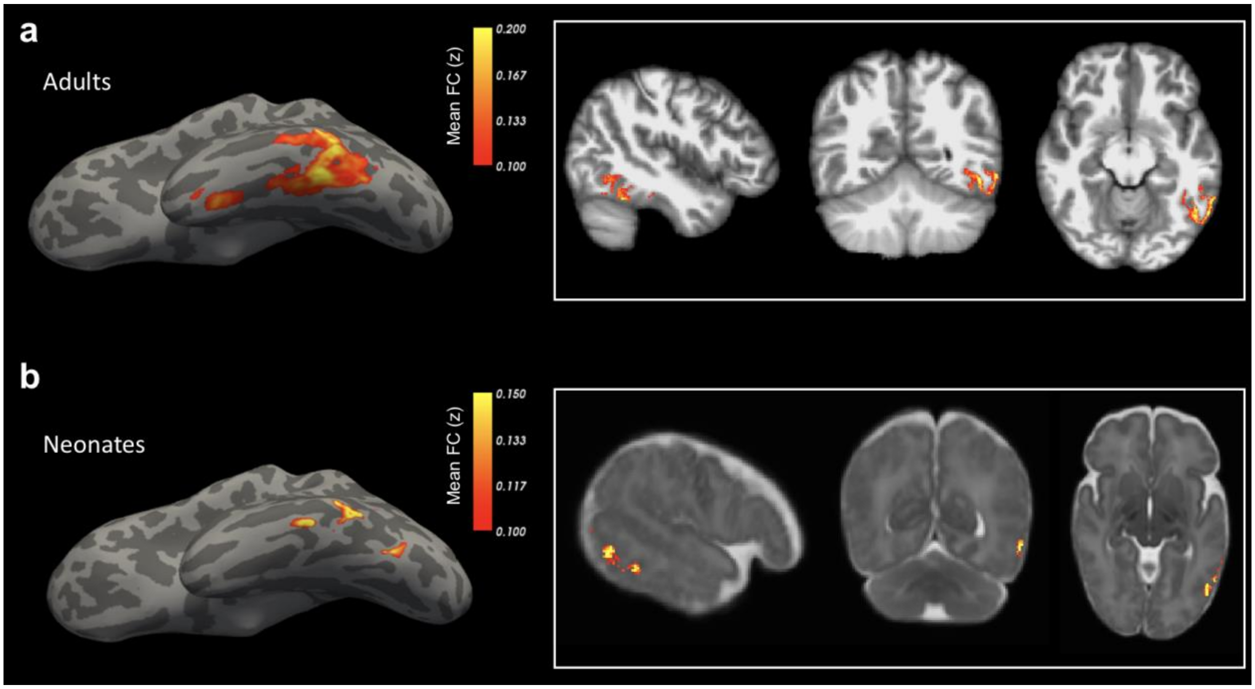

Supplementary Figure 3 | Averaged voxel-wise functional connectivity maps within the ventral temporal cortex (VTC) using language regions as the seed. Functional connectivity maps were calculated (fisher's z transformed, z(r)) for each individual and then averaged across individuals. **(a)** all adults' maps were registered to the Freesurfer CVS_avg35_inMNI152 brain, and the averaged map across adults was projected to the surface (left) and volume (right) in the template space. **(b)** all neonates' maps were registered to 40-week (gestational age) template brain[8], and the averaged map across neonates was overlaid on the neonate template volume (right). Note that we also projected neonates map to the adult template surface (left) with the caveat that potential distortions may at the voxel-wise level.

Supplementary Results 4. We broke up the language regions into frontal and temporal components and performed the same FC analyses as in the main paper but comparing VWFA connectivity to the target regions grouped by temporal and frontal. We found that the VWFA was more connected with temporal language regions than speech and A1 regions in both neonates (Speech: $t(39) = 6.22$, $p < 0.001$, Cohen's $d = 0.98$, corrected, 95% CI = [0.08, 0.16]; A1: $t(39) = 6.01$, $p < 0.001$, Cohen's $d = 0.95$, corrected, 95% CI = [0.11, 0.22]) and adults (Speech: $t(39) = 5.48$, $p < 0.001$, Cohen's $d = 0.87$, corrected, 95% CI = [0.09, 0.20]; A1: $t(39) = 7.16$, $p < 0.001$, Cohen's $d = 1.13$, corrected, 95% CI = [0.17, 0.31]; Supplementary Figure 4a). We also found that the VWFA connected more with frontal language regions than MD regions in both neonates ($t(39) = 4.51$, $p < 0.001$; Cohen's $d = 0.71$, corrected, 95% CI = [0.05, 0.13]) and adults ($t(39) = 9.71$, $p < 0.001$, Cohen's $d = 1.54$, corrected, 95% CI = [0.23, 0.35]; Supplementary Figure 4b).

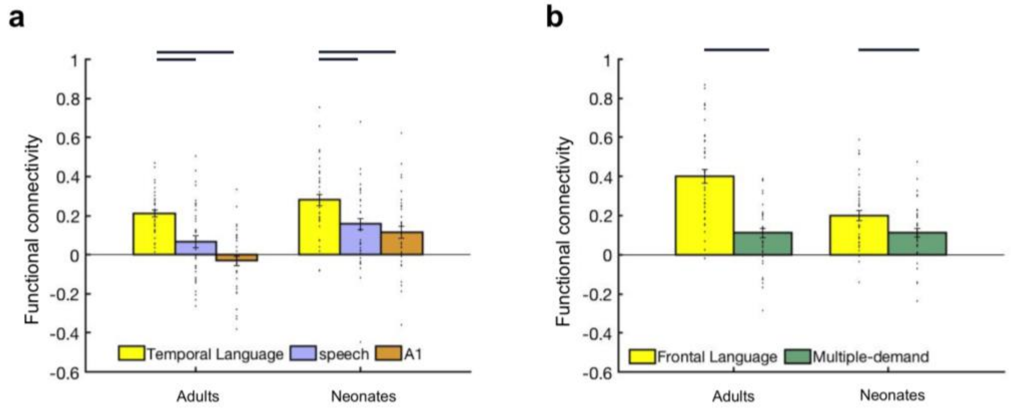

Supplementary Figure 4 | Functional connectivity between VWFA (seed) and temporal and frontal regions. (a) Mean FC between VWFA and regions in temporal (i.e., temporal language regions, speech, and A1). **(b)** Mean FC between VWFA and regions in frontal cortex (i.e., frontal language regions, multiple-demand regions). Connectivity values were Fisher z transformed. Error bars denote s.e.m. Individual data points (n = 40 for each age group) were shown for each category. Horizontal bars reflect significant post hoc paired t-tests $p < 0.05$, corrected.

We also performed the same parametric voxelwise analyses as done for the VTC in the main paper, but here we used the VWFA as the seed and characterized its FC to temporal and frontal cortex. Consistent with parcel-based analyses, when parametrically increasing threshold of FCs, we found that voxels revealed higher FC to VWFA located in temporal and frontal cortex that happen to be in language regions (vs. Speech and A1 regions in temporal, Supplementary Figure 5a; and vs. MD regions in frontal, Supplementary Figure 5b; heatmaps for VWFA's FC in frontotemporal cortices were also shown on representative adults and neonates surfaces, Supplementary Figure 5c).

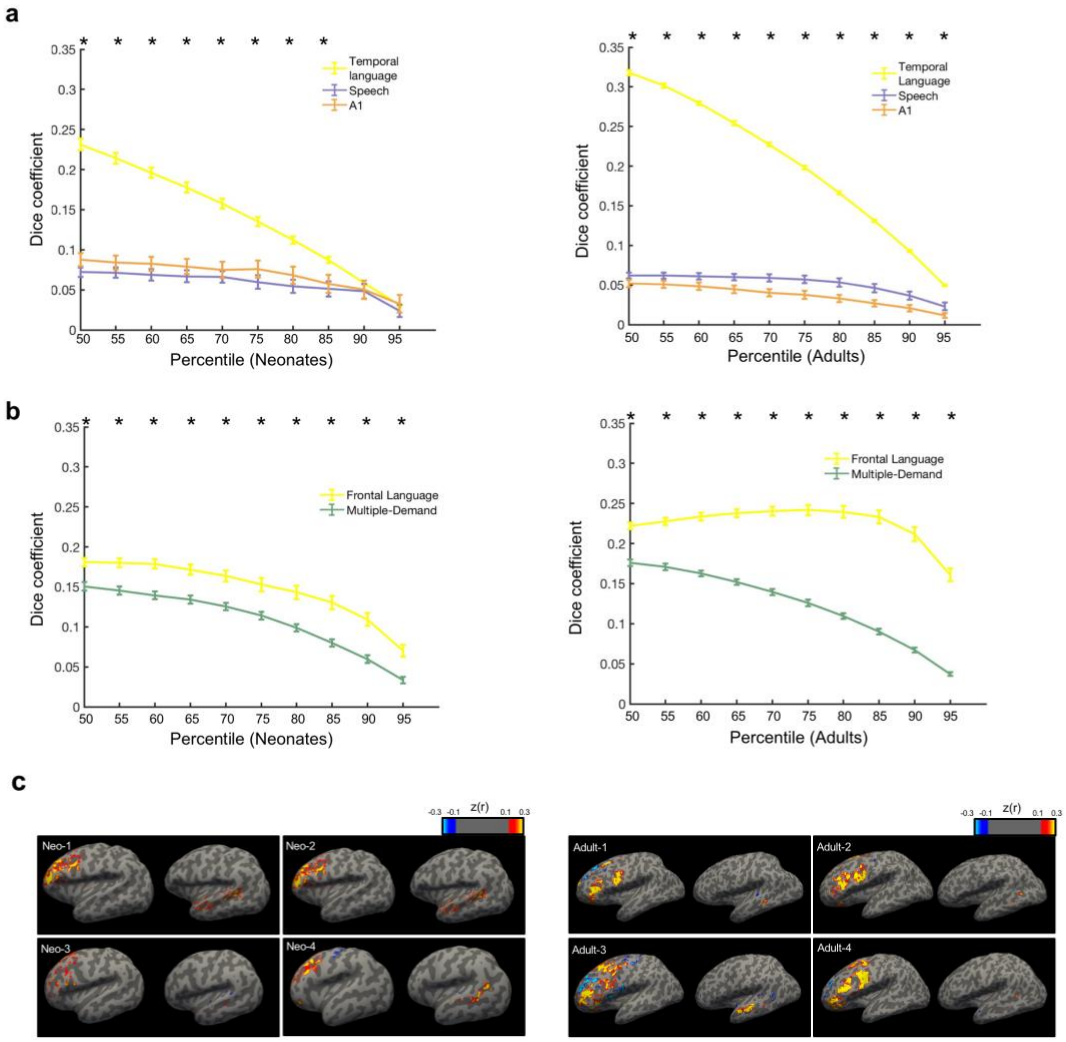

Supplementary Figure 5 | Voxel-wise analyses from VWFA to frontotemporal cortices. (a) As we parametrically increasing the threshold of FC within temporal cortex, we quantified how many of these voxels belonged in each functional category using *Dice coefficient*. Averaged FC (Fisher's z transformed) across neonates (n = 40; 50_{th}: z(r) = 0.19, p < 0.001; 95_{th}: z(r) = 0.39, p < 0.001); Average FC across adults

(n = 40; 50_{th}: z(r) = 0.17, p < 0.001; 95_{th}: z(r) = 0.42, p < 0.001). **(b)** As we parametrically increasing the threshold of FC within frontal cortex, we quantified how many of these voxels belonged in each functional category using Dice coefficient. Averaged FC across neonates (n = 40; 50_{th}: z(r) = 0.22, p < 0.001; 95_{th}: z(r) = 0.40, p < 0.001); Average FC across adults (n = 40; 50_{th}: z(r) = 0.16, p < 0.001; 95_{th}: z(r) = 0.39, p < 0.001). * denotes significant paired t-test (temporal/frontal language vs. averaged of other adjacent functional regions, p < 0.05, corrected). **(c)** Heatmaps for VWFA's connectivity within frontotemporal in representative neonates and adults, thresholded at z(r) greater than 0.1 (p < 0.001).

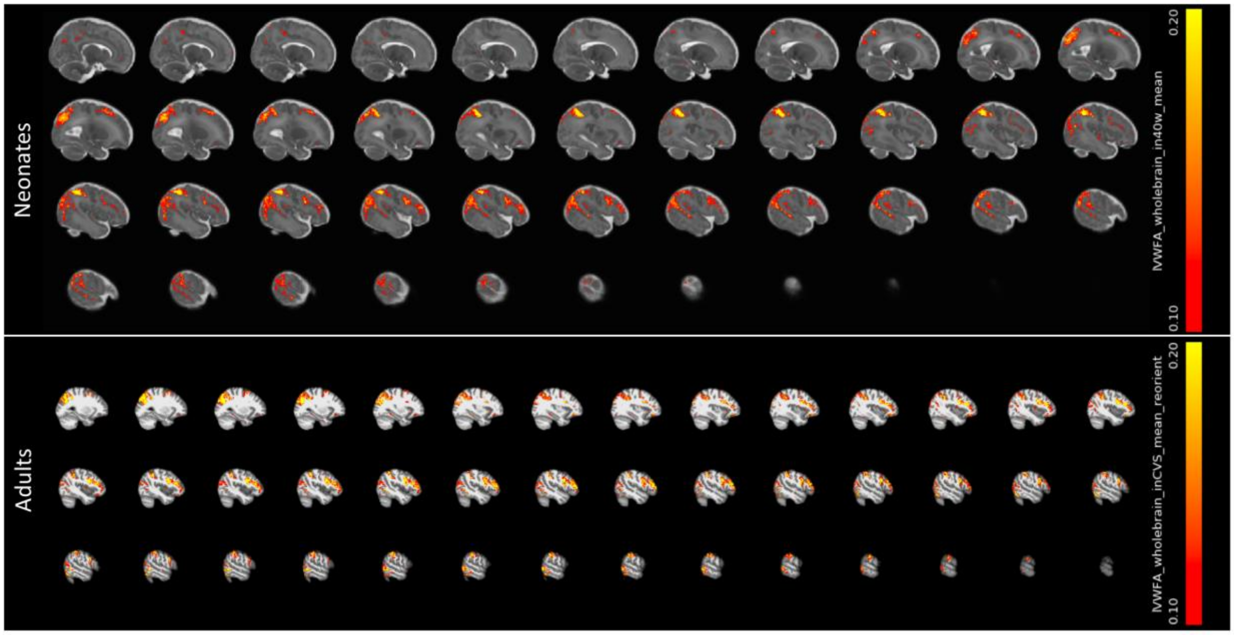

Supplementary Figure 6 | Averaged whole-brain functional connectivity maps of VWFA. VWFA whole-brain functional connectivity map was calculated (fisher's z transformed, z(r)) for each individual. All adults' maps were register to Freesurfer CVS_avg35_inMNI152 template brain, and all neonates' maps were registered to 40-week template brain[8]. The resultant averaged maps were threshold at 0.1< z(r) <0.3.

Supplementary Results 5. We obtained association test maps from a meta-analysis approach (Neurosynth, https://neurosynth.org) for all functions of interest (terms used: language (*1101 studies*), speech (*642*), primary auditory (*114*), faces (*864*), objects (*692*), place (*189*), visual word (*117*)) with a threshold of $z > 3$, and took the intersection between the functional parcels used in the main analyses with these Neurosynth-generated maps. These maps shared similar spatial locations with our functional parcels, but yield more conservative regions for most of functional categories (Supplementary Figure 7a and 7b, also see Supplementary Table 1 for sizes of these Neurosynth-overlapped parcels). Our main results remain the same with these new parcels (Supplementary Figure 7c-7e): language regions showed significantly higher connectivity to VWFA compared to other adjacent visual regions in adults (Faces: $t(39) = 8.46$, $p < 0.001$, Cohen's d = 1.34, 95% CI = [0.11, 0.19]; Scenes: $t(39) = 8.82$, $p < 0.001$, Cohen's d = 1.40, 95% CI = [0.16, 0.26]; Objects: $t(39) = 10.34$, $p < 0.001$, Cohen's d = 1.64, 95% CI = [0.13, 0.20], corrected). Similarly, neonates' language regions also connected more to VWFA than to faces ($t(39) = 7.11$, $p < 0.001$, Cohen's d = 1.12, 95% CI = [0.08, 0.15], corrected) and scenes ($t(39) = 7.19$, $p = 0.001$, Cohen's d = 1.29, 95% CI = [0.11, 0.20], corrected) but not objects ($t(39) = 1.98$, $p = 0.054$, Cohen's d = 0.31, 95% CI = [0, 0.07]). The difference of language connectivity to the VWFA vs. Objects was significantly larger in adults compared to neonates ($t(78) = 5.28$, $p < 0.001$, Cohen's d = 1.18, 95% CI = [0.08, 0.18]).When examining whether VWFA connects more to language regions than to other regions in the vicinity of language areas, we found that VWFA showed higher FC to temporal language regions than to speech (adults: $t(39) = 5.36$, $p < 0.001$, Cohen's d = 0.85, 95% CI = [0.08, 0.17]; neonates: $t(39) = 3.64$, $p = 0.001$, Cohen's d = 0.58, 95% CI = [0.03, 0.11], corrected) and A1 (adults: $t(39) = 6.95$, $p < 0.001$, Cohen's d = 1.10, 95% CI = [0.16, 0.29]; neonates: $t(39) = 3.94$, $p < 0.001$, Cohen's d = 0.62, 95% CI = [0.05, 0.16], corrected) and higher FC to frontal language regions to adjacent MD regions (adults: $t(39) = 4.47$, $p < 0.001$, Cohen's d = 0.71, 95% CI = [0.07, 0.20]; neonates: $t(39) = 4.46$, $p < 0.001$, Cohen's d = 0.71, 95% CI = [0.05, 0.14]) in both adults and neonates.

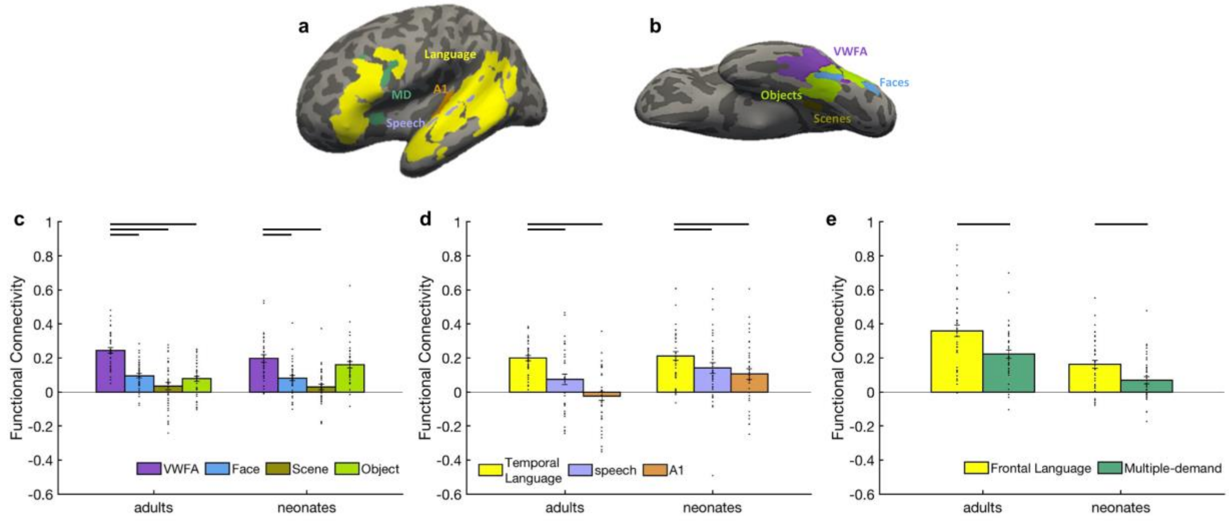

Supplementary Figure 7 | Functional connectivity results using new regions that intersected with Neurosynth-generated functional maps. (a) New parcels in temporal and frontal cortex. **(b)** New parcels in ventral temporal cortex. **(c)** Mean FC between language regions and ventral temporal visual regions (i.e., VWFA, faces, objects and scenes). **(d)** Mean FC between VWFA and regions in temporal cortex (i.e., temporal language regions, speech and A1)). **(e)** Mean FC between VWFA and regions in frontal cortex (i.e., frontal language regions, multiple-demand regions). Connectivity values were Fisher z transformed. Error bars denote s.e.m. Individual data points (n = 40 for each age group) were shown for each category. Horizontal bars reflect significant post hoc paired t-tests $p < 0.05$, corrected.

Supplementary Results 6. We used ANTs to register parcels to neonates and adults because it's commonly used in developmental studies [9-11]. We manually checked the registration results to ensure that functional parcels were in the relatively right locations (see Supplementary Figure 8a for example registration output for a representative adult and neonate). Next, for a quantitative analysis, we compared our registration accuracy to published registration methods used in a recent and comparable neuroimaging study of infants, where functional parcels were again defined based on fMRI data in a group of adults (and in fact using the same atlas/parcels that we are using here), and then registered to the infant brain but using FLIRT (FMRIB's Linear Image Registration Tool from FSL) instead of ANTs[12, 13]. We took the binary gray matter mask from CVS average-35 MNI152 template brain and then registered it to individual's native space with both ANTs and FLIRT. The registration result was compared to each individual's own binary gray matter mask. We found that in general, both methods yield high accuracy (over 90%), but ANTs significantly outperformed FLIRT (t(39)=6.21, p < 0.001, Cohen's d=0.62, 95% CI=[0.006, 0.01]; Supplementary Figure 8b).

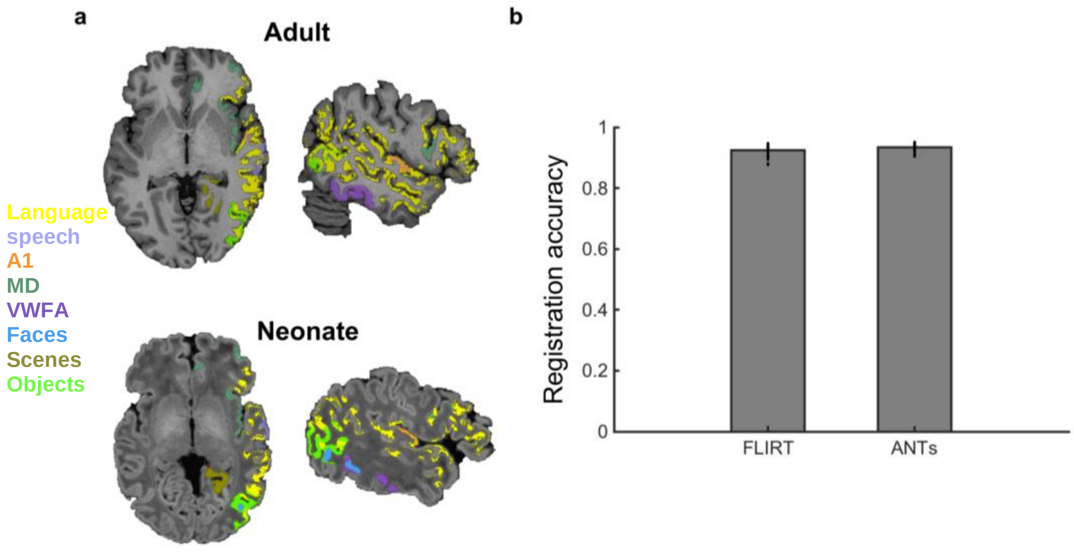

Supplementary Figure 8 | Registration results. (a) Registration results for all parcels from 8 categories for a representative adult (top) and neonate (bottom). **(b)** Comparing registration accuracy of binary gray matter mask using FLIRT and ANTs. ANTs significantly outperformed FLIRT (n = 40; t(39)=6.21, p < 0.001, Cohen's d = 0.62, 95% CI = [0.006, 0.01]).

Supplementary Results 7. The VWFA parcel used in the main analyses was created from the probabilistic map of words vs. line-drawing objects. This definition allowed us to capture the spatial variability of word-selective voxels across individuals. Here, we further restricted our definition of the VWFA to the posterior proportion to address the potential biases (e.g., localizers, sizes, and locations) of the VWFA used here. Specifically, by selecting the most posterior third of the current VWFA parcel, we created a VWFA-p which corresponding to the coordinates of the VWFA that had been reported in the previous literature[14, 15]. Our results remained the same using this more conservative definition of the VWFA (Supplementary Figure 9 and Supplementary Table 2 & 3).

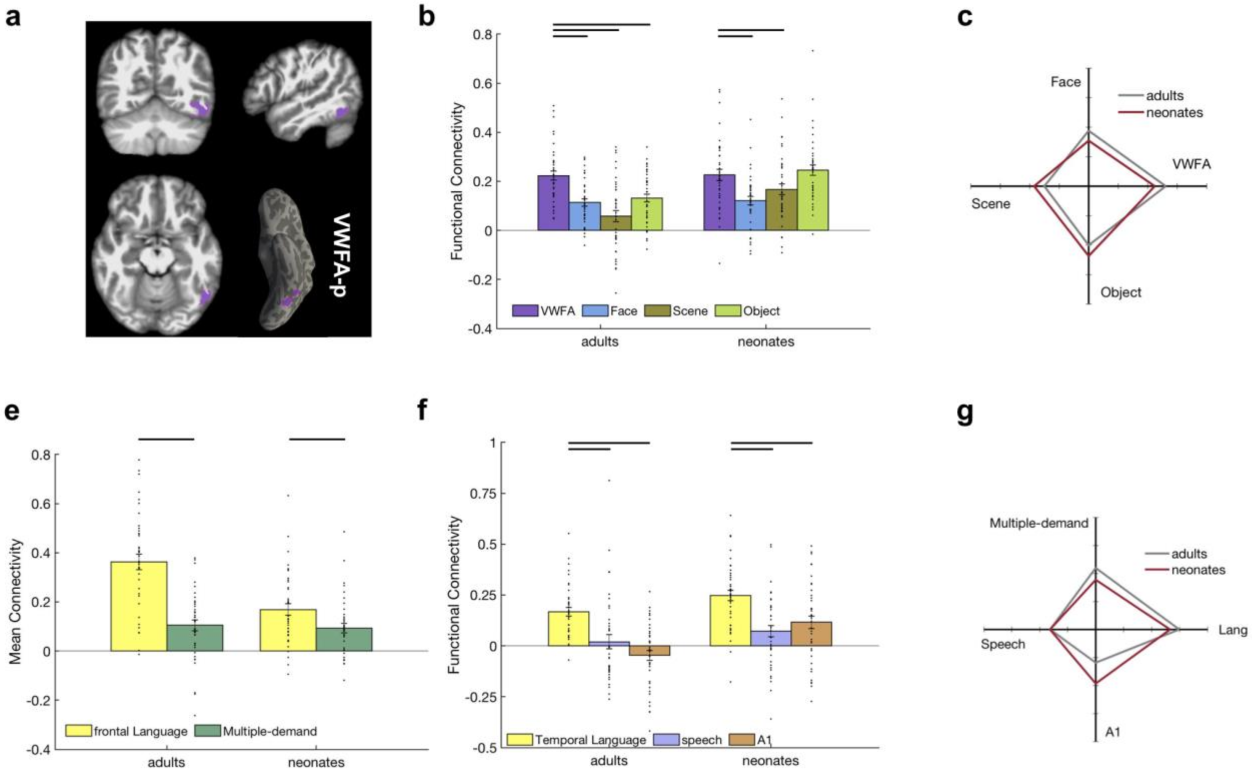

Supplementary Figure 9 | Functional connectivity results using VWFA-p. (a) The VWFA-p used here was shown in volume and surface. **(b)** Mean FC between language regions and ventral temporal visual regions (i.e., VWFA-p, faces, objects and scenes). **(c)** FC fingerprint of language regions. Connectivity values were mean-centered and averaged within each of the four categories to plot the relative patterns for the adult (n = 40) and neonate groups (n = 40). **(d)** Mean FC between VWFA-p and regions in temporal cortex (i.e., temporal language regions, speech and A1)). **(e)** Mean FC between VWFA-p and regions in frontal cortex (i.e., frontal language regions, multiple-demand regions). Connectivity values were Fisher z transformed. Error bars denote s.e.m. Individual data points (n = 40 for each age group) were shown for each category. Horizontal bars reflect significant post hoc paired t-tests $p < 0.05$, corrected.

Supplementary Table 1. Information of parcels used in the current study.

Category	How to define	Regions (Left)	Coordinate (CVS_avg35_inMNI152 template space)			Size in mm^3 (original/within Neurosynth)		Source (number of subjects)
						Adults	Neonates	
Words	words vs. objects	VWFA	174	165	99	4688/3632	1163/815	Saygin et al., 2016 (20)
Face	Faces vs. objects	FFA	168	162	90	352/312	99/80	Julian et al., 2012 (40)
		OFA	164	154	65			
Scene	Scenes vs. obj	PPA	149	151	94	2552/536	616/60	
Object	Obj vs. scr.obj	LO	168	140	70	3608/2560	994/626	
		PFS	163	161	94			
Language	Engl vs. nonsn	*Temporal*						Fedorenko et al., 2010 (25)
		MidPostTemp	184	137	107	5032/1976	845/328	
		PostTemp	177	132	83			
		MidAntTemp	184	159	128			
		AntTemp	179	162	147			
		AngG	166	118	69			
		Frontal						
		IFG	174	123	161	4016/2648	676/427	
		IFGorb	169	149	182			
Multiple-demand	Hard vs. easy	SMA	134	87	145	2760/336	388/40	Fedorenko et al., 2013 (40)
		ACC	134	134	177			
		IFGorb	175	132	154			
		MFGorb	159	156	195			
		Insula	161	145	148			
Speech	bisyllabic pseudowords vs. baseline	within STG	189	149	126	1256/1256	209/209	Basilakos et al., 2018 (20)
A1	Anatomically defined	Primary auditory cortex	173	140	127	744/704	119/109	Desikan et al., 2006

* Engl, English sentences; nonsn, non-word sentences; Obj, objects; src.obj, scrambled objects; VWFA, visual word form area; FFA, fusiform face area; OFA, occipital face area; PPA, parahippocampal place area; LO, lateral occipital; PFS, posterior fusiform sulcus; AntTemp, anterior temporal lobe; MidAntTemp, middle-anterior temporal lobe; MidPostTemp, middle-posterior temporal lobe; PostTemp, posterior temporal lobe; AngG, angular gyrus; IFG, interior frontal gyrus; IFGorb, orbital IFG; MFGorb, orbital part of the middle frontal gyrus, IFGop, opercular part of the inferior frontal gyrus; SMA, supplementary motor area; ACC, anterior/mid cingulate cortex; STG, superior temporal gyrus.

Supplementary Table 2. Comparisons between language regions connectivity to VWFA-p vs. other visual regions.

Seed: Language regions				
Adults	t	df	*p*_corrected	Cohen's d
VWFA-p vs. Face	5.96	39	<0.001	0.94
VWFA-p vs. Scene	6.90	39	<0.001	1.09
VWFA-p vs. Object	5.38	39	<0.001	0.85
Face vs. Scene	2.18	39	0.047	0.34
Face vs. Object	-1.19	39	0.262	-0.19
Scene vs. Object	-3.92	39	0.001	-0.62
Neonates	t	df	*p*_corrected	Cohen's d
VWFA-p vs. Face	5.19	39	<0.001	0.82
VWFA-p vs. Scene	2.32	39	0.038	0.37
VWFA-p vs. Object	-0.98	39	0.333	-0.15
Face vs. Scene	-2.07	39	0.055	-0.33
Face vs. Object	-8.18	39	<0.001	-1.29
Scene vs. Object	-4.18	39	<0.001	-0.66

Supplementary Table 3. Comparisons between VWFA-p connectivity to language regions vs. adjacent regions in temporal and frontal cortices.

Seed: VWFA-p				
Adults	t	df	*p*_corrected	cohen's d
TempLang-Speech	5.76	39	<0.001	0.91
TempLang-A1	6.97	39	<0.001	1.10
Speech-A1	1.61	39	0.132	0.25
FrontLang-MD	10.74	39	<0.001	1.70
Neonates	t	df	*p*_corrected	Cohen's d
TempLang-Speech	7.99	39	<0.001	1.26
TempLang-A1	4.80	39	<0.001	0.76
Speech-A1	-1.23	39	0.226	-0.19
FrontLang-MD	4.01	39	<0.001	0.63

Supplementary References

1. Saygin, Z.M., *et al.* Connectivity precedes function in the development of the visual word form area. *Nat. Neurosci.* **19**, 1250 (2016).
2. Fedorenko, E., Hsieh, P.-J., Nieto-Castañón, A., Whitfield-Gabrieli, S. & Kanwisher, N. New method for fMRI investigations of language: defining ROIs functionally in individual subjects. *J. Neurophysiol.* **104**, 1177-1194 (2010).
3. Overath, T., McDermott, J.H., Zarate, J.M. & Poeppel, D. The cortical analysis of speech-specific temporal structure revealed by responses to sound quilts. *Nat. Neurosci.* **18**, 903 (2015).
4. McDermott, J.H. & Simoncelli, E.P. Sound texture perception via statistics of the auditory periphery: evidence from sound synthesis. *Neuron.* **71**, 926-940 (2011).
5. Fedorenko, E., Duncan, J. & Kanwisher, N. Broad domain generality in focal regions of frontal and parietal cortex. *Proc. Natl. Acad. Sci. U.S.A.* **110**, 16616-16621 (2013).
6. Pitcher, D., Dilks, D.D., Saxe, R.R., Triantafyllou, C. & Kanwisher, N. Differential selectivity for dynamic versus static information in face-selective cortical regions. *Neuroimage.* **56**, 2356-2363 (2011).
7. Julian, J.B., Fedorenko, E., Webster, J. & Kanwisher, N. An algorithmic method for functionally defining regions of interest in the ventral visual pathway. *Neuroimage.* **60**, 2357-2364 (2012).
8. Serag, A., *et al.* Construction of a consistent high-definition spatio-temporal atlas of the developing brain using adaptive kernel regression. *Neuroimage.* **59**, 2255-2265 (2012).
9. Wang, H. & Yushkevich, P. Multi-atlas segmentation with joint label fusion and corrective learning—an open source implementation. *Front Neuroinform.* **7**, 27 (2013).
10. Menze, B.H., *et al.* The multimodal brain tumor image segmentation benchmark (BRATS). *IEEE Trans Med Imaging.* **34**, 1993-2024 (2014).
11. Avants, B.B., *et al.* The Insight ToolKit image registration framework. *Front Neuroinform.* **8**, 44 (2014).
12. Deen, B., *et al.* Organization of high-level visual cortex in human infants. *Nat Commun.* **8**, 13995 (2017).
13. Kamps, F.S., Hendrix, C.L., Brennan, P.A. & Dilks, D.D. Connectivity at the origins of domain specificity in the cortical face and place networks. *Proc. Natl. Acad. Sci. U. S. A.* **117**, 6163-6169 (2020).
14. Glezer, L.S. & Riesenhuber, M. Individual variability in location impacts orthographic selectivity in the "visual word form area". *J Neurosci.* **33**, 11221-11226 (2013).
15. Vogel, A.C., Petersen, S.E. & Schlaggar, B.L. The left occipitotemporal cortex does not show preferential activity for words. *Cereb. Cortex.* **22**, 2715-2732 (2012).
